# Lower local recurrence rate after robot-assisted thoracoscopic esophagectomy than conventional thoracoscopic surgery for esophageal cancer

**DOI:** 10.1038/s41598-021-86420-x

**Published:** 2021-03-24

**Authors:** Satoru Motoyama, Yusuke Sato, Akiyuki Wakita, Yushi Nagaki, Hiromu Fujita, Ryohei Sasamori, Kohei Kemuriyama, Shinogu Takashima, Kazuhiro Imai, Yoshihiro Minamiya

**Affiliations:** 1 Esophageal Surgery, Akita University Hospital, Akita University School of Medicine, 1-1-1 Hondo, Akita, 010-8543 Japan; 2 Comprehensive Cancer Control, Akita University Graduate School of Medicine, Akita, Japan; 3 Thoracic Surgery, Akita University Graduate School of Medicine, Akita, Japan

**Keywords:** Surgical oncology, Oesophageal cancer

## Abstract

The oncological advantages of robot-assisted thoracoscopic esophagectomy (RATE) over conventional thoracoscopic esophagectomy (TE) for thoracic esophageal cancer have yet to be verified. In this study, we retrospectively analyzed clinical data to compare the incidences of recurrence within the surgical field after RATE and TE as an indicator of local oncological control. Among 121 consecutive patients with thoracic esophageal or esophagogastric junction cancers for which thoracoscopic surgery was indicated, 51 were treated with RATE while 70 received TE. The number of lymph nodes dissected from the mediastinum, duration of the thoracic portion of the surgery, and morbidity due to postoperative complications did not differ between the two groups. However, the rate of overall local recurrence within the surgical field was significantly (*P* = 0.039) higher in the TE (9%) than the RATE (0%) group. Lymph node recurrence within the surgical field occurred in left recurrent nerve, left tracheobronchial, left main bronchus and thoracic paraaortic lymph nodes, which were all difficult to approach to dissect. The other two local failures occurred around the anastomotic site. This study indicates that using RATE enabled the incidence of recurrence within the surgical field to be reduced, though there were some limitations.

## Introduction

About 20 years have passed since the first experiences with the innovative transthoracic robot-assisted thoracoscopic esophagectomy (RATE) were reported^1–3^. Theoretically, the four arms employed with RATE have sufficient dexterity to increase operative precision and maneuverability within the narrow space of the mediastinum. In addition, it was reported that RATE was feasible and safe, and surgeons could learn to use it within a relatively short period^4–7^. Investigators then began comparing the intraoperative and short-term outcomes between their early cases with RATE and those with conventional thoracoscopic esophagectomy (TE)^8–11^. RATE reportedly reduced blood loss, the incidence of vocal cord palsy, and hospital stay duration as compared to TE, though the operations took longer and had a significantly higher financial cost in several cohorts. Regarding intraoperative oncological factors, RATE enabled dissection of a higher number of lymph nodes along the left recurrent laryngeal nerve (RLN) without increasing morbidity^10,11^. We also reported that the extent of lymph node dissection around the left RLN in the left lateral decubitus position was more powerful with RATE than TE^12^. On the other hand, there have been few reports demonstrating definitive a mid- or long-term oncological benefit with RATE. Thus, further evidence showing an oncological benefit of RATE over TE is urgently needed. To add to the available data, in the present study we assessed recurrence within the surgical field with RATE in our early cases as an indicator of the ability to maintain local control for mid-term oncological benefit.

## Results

Between December 2014 and April 2020, 51 consecutive patients who had undergone RATE with R0 resection using the da Vinci S, Si or Xi Surgical System were enrolled in this retrospective study of our early experience. All operations using the da Vinci Surgical System were performed by a consultant surgeon (SM). During the same period, 70 patients underwent conventional TE with R0 resection in a left lateral position. The operations were performed by five surgeons, including two certified esophageal surgeons and three noncertified surgeons. Thus, the early experiences of three noncertified surgeons are included. Patients were observed for a median of 20 months (range, 5–77 months) after esophagectomy in the RATE group and 41 months (range, 6–72 months) in the TE group.

The characteristics of the patients and clinical stages, histological type of the cancers, and preoperative treatments in the RATE and TE groups are summarized in Table 1. There were no significant differences between the two groups with respect to age, sex, tumor location, tumor depth, lymph node metastasis, number of involved nodes, distant metastasis, clinical stage or histological type. The percentages of patients who received neoadjuvant therapy also did not differ.

**Table 1:** Characteristics of the patients in the RATE and TE groups.

	RATE (N = 51)	TE (N = 70)	*P*
Age, years, median (range)	65 (44–80)	67 (41–85)	0.212
**Sex, n (%)**			
Male	45 (88%)	60 (86%)	0.790
Female	6 (12%)	10 (14%)	
**Tumor location, n (%)**			
Upper	8 (16%)	11 (16%)	0.839
Middle	20 (39%)	30 (43%)	
Lower	16 (31%)	23 (33%)	
Esophagogastric junction	7 (14%)	6 ( 9%)	
**cT, n(%)**			
T1	18 (35%)	23 (37%)	0.494
T2	9 (18%)	6 (9%)	
T3	23 (45%)	37 (53%)	
T4	1 ( 2%)	1 (1%)	
**cN, n (%)**			
0	23 (45%)	34 (49%)	0.717
1–2	28 (55%)	36 (51%)	
Number of involved nodes, median (range)	1 ( 0–6)	1 ( 0–4)	0.560
**cStage, n (%)**			
IA	13 (25%)	23 (33%)	0.448
IB	5 (10%)	3 ( 4%)	
IIA	5 (10%)	8 (11%)	
IIB	9 (18%)	4 ( 6%)	
IIIA	9 (18%)	18 (26%)	
IIIB	6 (12%)	9 (13%)	
IIIC	1 ( 2%)	1 ( 1%)	
IV	3 ( 6%)	4 ( 6%)	
**Histological type of cancer**			0.200
Squamous cell carcinoma	43 (84%)	64 (91%)	
Adenocarcinoma	6 (12%)	6 (9%)	
Other	2 (4%)	0 (0%)	
**Neoadjuvant therapy, n (%)**			
Chemoradiotherapy	27 (53%)	31 (44%)	0.311
Chemotherapy	2 (4%)	7 (10%)	
Endoscopic resection	1 (2%)	5 (7%)	
None	21 (41%)	27 (39%)	

The operation fields and times for the thoracic portion of the surgery were nearly the same in the two groups; however, blood loss during the thoracic surgery was significantly lower in the RATE group than the TE group (Table 2). The number of dissected lymph nodes did not differ between the two groups. Morbidity due to postoperative complications, such as pneumonia (Uniform Pneumonia Score (UPS) ≥ 2, with at least 1 point being assigned due to infiltrative findings on pulmonary radiography), anastomotic leakage (Type ≥ I in Esophageal Complications Consensus Group (ECCG) standardized definitions), and recurrent nerve palsy (Type ≥ I in ECCG standardized definitions) also did not differ between the two groups (Table 2 and Supplement Table)^13,14^.

**Table 2:** Surgical outcomes of patients in the RATE and TE groups.

	RATE (N = 51)	TE (N = 70)	*P*
**Area of lymph node dissection, n (%)**			
Mediastinum and upper abdomen (2-field)	4 (8%)	6 (9%)	0.664
Bilateral neck, mediastinum and upper abdomen (3-field)	40 (78%)	58 (83%)	
Lower mediastinum and upper abdomen	7 (14%)	6 ( 9%)	
**Reconstruction**			
Gastric tube	49 (96%)	67 (96%)	0.921
Pedicled colon	2 (4%)	3 (4%)	
**Approach for the abdominal portion**			
Open	27 (53%)	47 (67%)	0.003*
Hand-assisted laparoscopic	14 (27%)	19 (27%)	
Total laparoscopic	1 (2%)	4 (6%)	
Robot-assisted laparoscipic	9 (18%)	0 ( 0%)	
**Operation time (min), median (range)**			
All	646 (485–852)	606 (410–975)	0.201
Thoracic portion	297 (188–457)	298 (144–580)	0.753
**Blood-loss (ml), median (range)**			
All	407 (43–2355)	417 (129–3366)	0.684
Thoracic portion	91 (0–623)	148 (12–1858)	0.004*
**Number of dissected lymph nodes, median (range)**			
All	52 (14–104)	54 (7–97)	0.325
Mediastinal	21 (0–45)	19 (0–68)	0.741
**Pneumonia (UPS), n (%)**			0.365
Negative	43 (84%)	54 (77%)	
Positive	8 (16%)	16 (23%)	
**Anastomotic leak (Type ≧ I), n (%)**			1.000
Negative	46 (90%)	63 (90%)	
Positive	5 (10%)	7 (10%)	
**Recurrent laryngeal nerve palsy (Type ≧ I), n (%)**			0.414
Negative	39 (76%)	48 (67%)	
Positive	12 (24%)	22 (33%)	
Death in hospital	0 (0%)	0 (0%)	1.000
Death within 90 days after esophagectomy	0 (0%)	0 (0%)	1.000

*UPS* uniform pneumonia score.

*Statistically significant difference.

**Figure 1:**
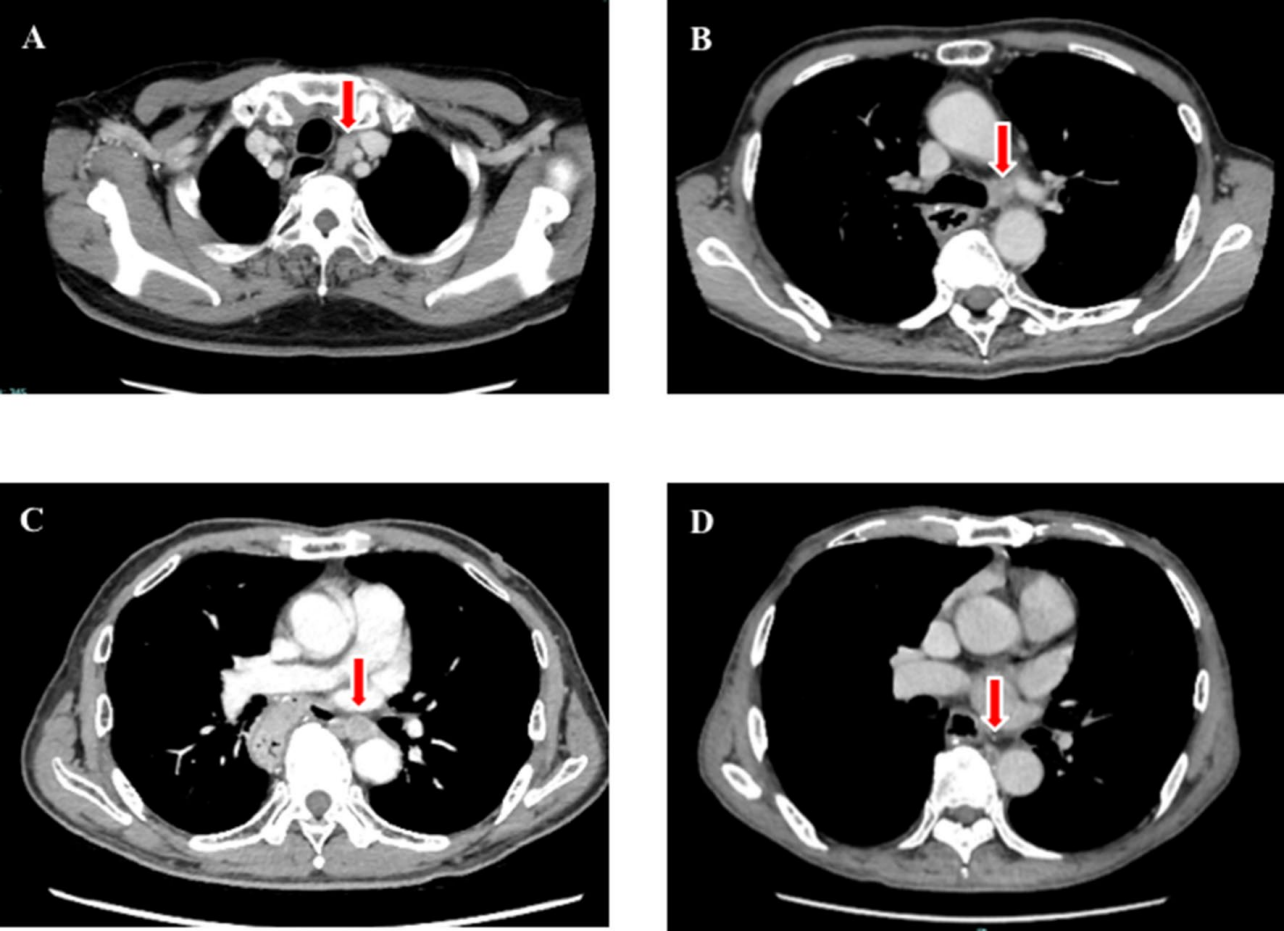
Contrast-enhanced computed tomography images of recurrent lymph nodes: (**a**) left recurrent laryngeal nerve lymph node; (**b**) left tracheobronchial lymph node; (**c**) left main bronchus lymph node; (**d**) thoracic paraaortic lymph node.

**Figure 2:**
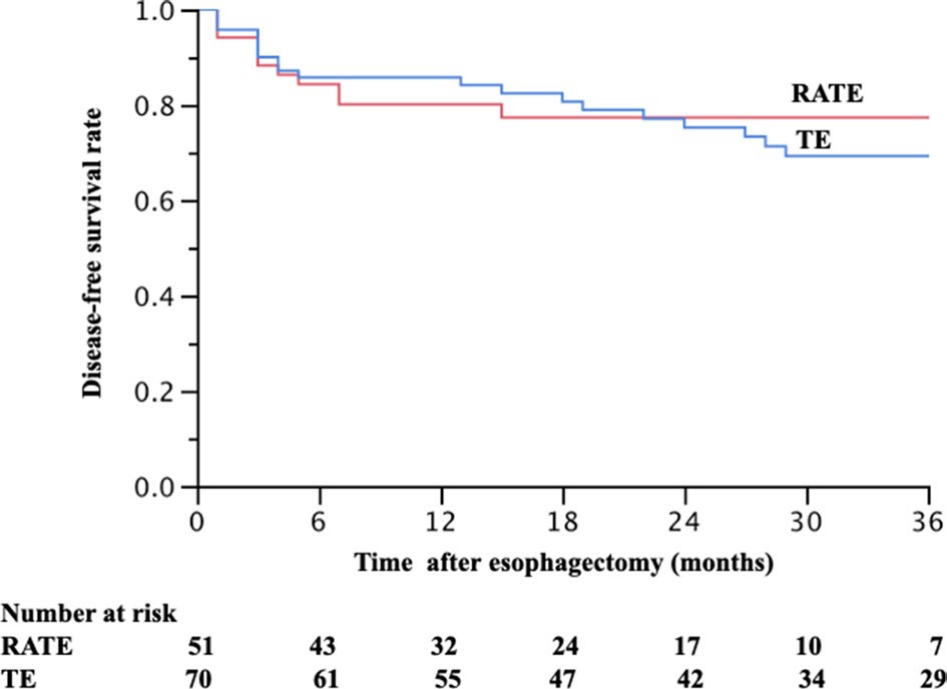
Disease-free survival curve in the patients received robot-assisted thoracoscopic esophagectomy and conventional thoracoscopic esophagectomy. *RATE* robot-assisted thoracoscopic esophagectomy, *TE* thoracoscopic esophagectomy.

During the observational period, recurrence rates were 22% and 27% in the RATE and TE group, respectively. Interestingly, the lymph node recurrence rate within the surgical field was 6% (4 patients) in the TE group, but was 0% in the RATE group. Lymph node recurrences involved a left recurrent nerve lymph node (106recL: Lymph node number according to the Japanese Classification of Esophageal Cancer, 11th edition), a left tracheobronchial lymph node (106tbL), a left main bronchus lymph node (109L), and a thoracic paraaortic lymph node (112ao), which were difficult to approach for lymph node dissection (Fig. 1)^15,16^]. In addition, two patients had recurrences around the anastomotic site. Overall, the local recurrence rate was 9% (6 patients) in the TE group, which was significantly (*P* = 0.039) higher than the 0% recurrence rate in the RATE group (Table 3). Among patients showing recurrence, 3 received neoadjuvant chemoradiotherapy, while the other 3 received up-front surgery. In the RATE group, all recurrence sites were distant fields (distant organ or distant lymph node). On the other hand, the ratio of surviving patients and the disease-free survival (DFS) rates did not differ between the two groups (Table 3 and Fig. 2).

**Table 3:** Recurrence and survival among patients in the RATE and TE groups.

	Robot (N = 51)	Thoracoscopy (N = 70)	*P*
**Recurrence**			0.393
None	40 (78%)	51 (73%)	
Recurred	11 (22%)	19 (27%)	
**Lymph node recurrence within the surgical field**			0.137
None	51 (100%)	66 (94%)	
Recurred within the surgical field	0 (0%)	4 (6%)	
**Overall local recurrence (including anastomotic site)**			0.039*
None	51 (100%)	64 (91%)	
Recurred within a local field	0 (0%)	6 (9%)	
**Recurrence pattern**			0.189
None	40 (78%)	51 (73%)	
Local field	0 (0%)	6 (9%)	
Distant lymph node	4 (8%)	5 (7%)	
Distant organs	7 (14%)	8 (11%)	
**Survival**			0.947
Alive	40 (78%)	55 (79%)	
Esophageal cancer-specific death	8 (16%)	10 (14%)	
Death from other diseases	3 (6%)	5 (7%)	
**Disease-free survival rate**			0.697^#^
1-year survival rate	76.5%	81.4%	
2-year survival rate	74.1%	71.8%	
3-year survival rate	74.1%	64.7%	

*Statistically significant difference.

^#^Log-rank test.

## Discussion

This study revealed several interesting results. First, the local recurrence rate was 9% in the TE group, which was significantly (*P* = 0.039) higher than the 0% recurrence rate in the RATE group. Second, in the TE group recurrence in mediastinal lymph nodes was in a region that was difficult to approach for lymph node dissection. By contrast, in the RATE group all recurrence sites were in distant fields (a distant organ or distant lymph node).

Thoracic esophageal cancer is one of the most aggressive cancers and is characterized by rapid clinical progression and a poor prognosis. Consequently, neoadjuvant chemo- or chemoradiation therapy is usually indicated, even in patients who will then undergo esophagectomy^17^. Indeed, the current international guidelines recommend combined treatments consisting of chemotherapy, radiotherapy and surgery for patients with localized advanced esophageal or esophagogastric cancer^18–21^. After neoadjuvant treatments, esophagectomy with extended lymph adenectomy in the neck, mediastinum and abdomen was performed as the main component of this curative and radical treatment strategy. Unfortunately, patients with esophageal cancer often have comorbidities and are in poor clinical condition as a result of advanced age, body weight loss, habitual alcohol use, smoking, poor respiratory function (chronic obstructive pulmonary disease), hypertension, and/or a history of prior cancer. As a result, postoperative complications are more frequent in these patients, even now. To decrease the invasiveness of the operation and postoperative complications while providing a survival benefit to these patients, surgeons have been applying minimally invasive techniques since the 1990’s. However, as Straatman et al. reported, the randomized controlled TIME Trial revealed that there were no differences in 3-year disease-free or overall survival between open transthoracic esophagectomy and minimally invasive TE^22^. With the rapid shift from conventional TE to RATE, we must rapidly produce oncological benefit with RATE as compared to TE or open transthoracic esophagectomy. To determine the long-term oncological benefit of RATE, a randomized trial testing whether RATE is superior to conventional TE is currently in progress and is targeted to 5-year overall survival as a primary endpoint^23^]. It will be several years before a conclusion is reached.

Regarding the intraoperative or short-term surgical outcomes with RATE, which will influence the oncological benefit, several reports, including ours, have demonstrated radical lymph node dissection around the left RLN, which is known to be a difficult field for lymph node dissection, without increasing left recurrent nerve palsy^10–12^. Moreover, several other powerful findings were also recently added. Yong et al. reported in 2019 that their RATE group (n = 280) yielded more lymph nodes along the RLN (4.8 vs. 4.1) with a shorter surgical duration for the thoracic portion than their TE (n = 372) group (85.0 vs. 102.9 min), but the incidence of RLN injury was higher in the RATE group (29.2% vs. 15.1%)^24^. Tagkalos et al. used propensity-matched analysis to assess consecutive patients with esophageal cancer undergoing modified Ivor Lewis esophagectomy. They reported that there was a trend toward improved lymphadenectomy with a shorter stay in the intensive care unit with RATE (n = 50) than TE (n = 50)^25^. In addition, Harbison et al. conducted a retrospective analysis with risk-adjustment using a nationally-validated database: the American College of Surgeons National Surgical Quality Improvement Program (ACS-NSQIP). They found that surgical outcomes did not significantly differ between the RATE (n = 100) and TE (n = 625) groups with respect to the incidence of 30-day postoperative mortality or overall morbidity^26^. However, van der Horst et al. reported that among patients with lymph node-positive thoracic esophageal cancer in the superior mediastinum, RATE was associated with higher mortality and morbidity^27^.

Regarding the mid-term oncological benefits of RATE, Yong et al. reported that RATE was associated with a lower rate of mediastinal lymph node recurrence (2.0% vs. 5.3%) (*P* = 0.044), but overall and disease-free survival did not differ between the two cohorts^24^. Our present study strengthens and clarifies the observational findings on the mid-term oncological benefits of RATE. Although the extent of the dissection and number of lymph nodes dissected from the mediastinum did not differ between the RATE and TE groups, local recurrence, including lymph node recurrence within the surgical fields, was significantly higher with conventional TE than RATE. RATE enables surgeons to precisely maneuver and produce good oncological outcomes. This appears to reflect the advantages of RATE, which include a 3D self-controlled magnified view enabling better visualization of this narrow area and the ability to offer adequate depth perception. In addition, use of a self-controlled third arm and a tremor filtering function enabled us to achieve fine tension and countertraction during dissection in this narrow area. However, such recurrences are not always due to surgical failure, as these recurrences occurred a considerable time (3–24 months) after esophagectomy. Although the rate of local failure was higher in the TE group than the RATE group, the ratios of surviving patients and the DFS rates did not differ between the two groups. One reason for that is the majority of recurrences were in distant fields in both groups, and the rates of local recurrence were relatively low. In addition, local failures were treatable and had the possibility of cure. Indeed, we treated the six affected patients with additional surgical resection of the recurrent lymph node followed by chemoradiotherapy or definitive chemoradiotherapy, and three patients were completely cured with no further recurrence.

This study has several limitations. First, the study population was heterogeneous, the number of patients in the cohort was small, and the number of events was limited. Second, it was a nonrandomized comparative analysis, and there was considerable bias in the selection of RATE or TE as the surgical approach. Third, only short- and mid-term results were determined. To assess overall oncological benefit, we will need to follow these patients for a longer time. The presented result is extremely important, but it is only a preliminary report using our first case series with RATE. Further analysis is therefore necessarily.

In summary, our findings indicate that RATE enables a reduction in the incidence of local recurrence within the surgical field. However, this should be interpreted with caution due to the limitations of this study mentioned above.

## Methods

This study was approved by the Ethics Committee of Akita University Graduate School of Medicine (No. 1222). All methods were performed in accordance with the relevant guidelines and regulations. All participants provided informed consent and signed a human subject institutional review board consent form.

### Selection of approach of surgery

We began using RATE for patients with thoracic esophageal and esophagogastric junction cancer in December 2014. The procedure was indicated for all patients with thoracoscopically resectable cancers and with Eastern Cooperative Oncology Group performance status 0. Since April 2018, RATE has been covered by the health insurance system in Japan, which covers all of Japan’s citizens. It enables us to perform RATE for all patients; however, there are several licenses that the surgeon must obtain. Although in the present study all surgeries were performed by one team, the selection of approach (RATE vs. TE) for the thoracic part depended upon the operating surgeon. Between December 2014 and March 2018, RATE was selected by patients who desired to receive RATE, despite the lack of health insurance coverage; for the other patients, TE was performed with insurance coverage. The extent and fundamental technique used for dissection of the tumor and lymph nodes is same for both approaches. The abdominal surgeries were performed using an open, hand-assisted laparoscopic or a total laparoscopic approach in the TE group. In the RATE group, one surgeon added a robot-assisted laparoscopic approach to the other approaches for the abdominal surgery. The selection of the abdominal approach was also decided by the operating surgeon.

### Operative procedure

The patients were placed in the left lateral position under a combination of inhaled and intravenous anesthesia, and a double-lumen endotracheal tube was used for single-lung ventilation during the thoracic part of the surgery. With RATE, the right arm was raised 60° cranially to expose the right axillar fossa, then tilted 20° cranially and 15° ventrally. The assistant surgeons stood on the left side of the patient. The da Vinci trocars (8 mm) were inserted into the 2nd or 3rd intercostal space (ICS) on the anterior axillary line (AL), the 4th or 5th ICS on the middle AL, the 6th or 7th ICS on the middle AL, and the 9th or 10th ICS on the posterior AL. Generally, an additional trocar was inserted into the 4th ICS on the anterior AL for an assistant and insufflation of the thoracic cavity with CO_2_ (8 mmHg). We mainly used a forward-oblique viewing endoscope during the thoracic portion of the surgery. In TE, a 25-mm mini-thoracotomy or a 12-mm trocar insertion was performed in the 4th ICS on the anterior AL for an assistant. Four 10.5-mm trocars were inserted into the 4th ICS on the posterior AL for the operator’s left arm, the 5th ICS on the middle AL for the scope (for an assistant surgeon), the 7th ICS on the posterior AL for the operator’s right arm, and the 8th ICS on the anterior AL for an assistant. A forward-oblique viewing endoscope was used during the thoracic portion of the surgery. The thoracic portion of the operation was nearly the same in the RATE and TE groups.

For thoracic esophageal cancer, the operation was begun with the thoracic portion and incising of the mediastinal pleura on the dissected line. The arch of the azygos vein was divided and then ligated. The lymph nodes around the right RLN were dissected below the right subclavian artery. To dissect along the left RLN, trachea and main bronchus were displaced to the ventral side to enlarge the limited space to increase the range of movement of the surgical instrument. The thoracic duct was carefully preserved in T1-2 cancers, but was resected together with the tumor in T3 cancers. The bilateral pulmonary branches of the vagal nerves were preserved. The lower posterior mediastinal lymph nodes were dissected from the pericardium, left pleura, descending aorta, and diaphragm. For middle and lower thoracic esophageal cancers, the esophagus was divided at the level of the upper edge of the aortic arch by linear stapling; for upper thoracic esophageal cancers, it was divided in the supradiaphragmatic area. Next, the abdominal portion of the surgery and bilateral neck lymph node dissection were performed concurrently. Our standard strategy for lymph node dissection was 3-field lymphadenectomy (bilateral neck, mediastinal and upper abdominal lymph nodes) for thoracic esophageal cancer. This was followed by reconstruction using the stomach or pedicled colon and handsewn layer-to-layer anastomosis.

For esophagogastric junction cancers, the surgery was begun with the abdominal portion. This consisted of abdominal lymph node dissection in the perigastric region and areas around the celiac axis, common hepatic artery and splenic artery, and making a gastric roll using an open or a total laparoscopic approach, including a robot-assisted approach. The thoracic portion of this surgery began with incision of the mediastinal pleura on the dissected line. The arch of the azygos vein and thoracic duct were usually preserved. The lower and middle posterior mediastinal lymph nodes were dissected from the pericardium, left pleura, descending aorta, and diaphragm. The lymph nodes around the main bronchus were prophylactically dissected in nearly all patients. Using linear stapling, the esophagus was divided at the middle thoracic esophagus. The gastric tube was then pulled up into the intrathoracic space and reconstructed by making an anastomosis between the remaining esophagus and the gastric tube using linear or circular stapling.

### Clinical staging

The clinical staging, including diagnosis of lymph node metastasis, was defined at a conference attended by radiologists, physicians and surgeons according to the International Union Against Cancer tumor-node-metastasis (TNM) Classification of Malignant Tumors (seventh edition) based on findings from endoscopy, esophagography, contrast-enhanced computed tomography (CE-CT), and systematic [^18^F] fluorodeoxyglucose-positron emission tomography/computed tomography (FDG-PET/CT)^15,16,20,21^. Regional nodes were considered positive for malignancy when they were positive in FDG-PET/CT (the maximum standardized uptake value; SUVmax ≥ 2.5) and/or round or ovoid shaped with short axes ≥ 8 mm in thin-sliced CE-CT.

### Follow-up program

The post-surgical follow-up program consisted of blood tests, including those for tumor makers (squamous cell carcinoma antigen and carcinoembryonic antigen), and neck/chest/abdominal CE-CT every 4 months for up to 3 years, then every 6 months for up to 5 years. Upper gastrointestinal endoscopy was done yearly. FDG-PET/CT was used when recurrence was suspected. As a general rule, the patients visited the hospital once every 2 months for at least 5 years after their surgery.

### Outcomes

The intraoperative oncological surgical outcomes (operation time, estimated blood loss, the number of lymph nodes dissected from the mediastinum) and short- and midterm outcomes (incidence rate of postoperative complications, rate of local recurrence within surgical fields, and its site, patterns, and 3-year DFS rates) were compared between the two groups. The operating time for the thoracic portion of the surgery was defined as the time from the start of chest incision through closure of the trocar sites in the chest. Blood loss was estimated by weighing the suctioned blood and gauze pieces with absorbed blood. Surgical complications were evaluated using the ECCG standardized definitions^14^. Anastomotic leakage was observed using esophagography on postoperative day 8 and counted when it was Type ≥ I according to the ECCG definitions. Recurrent nerve palsy was observed using bronchoscopy on postoperative day 2 and counted when it was Type ≥ I according to the ECCG definitions. Postoperative pneumonia was scored according to the UPS and was evaluated as positive when UPS ≥ 2 with at least 1 point being assigned due to infiltrative findings on pulmonary radiography^13^. The other surgical complications were evaluated using the Clavien–Dindo classification^28^.

Local recurrences were confirmed using both CE-CT and FDG-PET/CT without pathological diagnosis, excluding patients who received surgical resection. Regarding local recurrence around the anastomotic site, we cannot confirm whether it was due to infiltration from a lymph node recurrence to the anastomotic site or from expansion of intramural metastasis in the esophagus or from the stomach, or to invasion of the anastomosis after regrowth of residual cancer cells around the anastomotic site. This is because intraoperative frozen sections showed these patients to be cancer free at the margin, and a clearly epithelial lesion was not seen at the time of recurrence. DFS was measured as the period from esophagectomy to the date of confirmed recurrence, death (whichever happened first), or the date of the investigators' last note of disease-free status on November 21, 2020.

### Data analysis

We retrospectively analyzed clinical data. Continuous variables are presented as medians (minimum–maximum), and differences between the two groups were analyzed using the Mann–Whitney-U test. Categorical data were analyzed using Pearson’s Chi square test or Fisher’s exact probability test. Overall survival was characterized using Kaplan–Meier curves. DFS curve was compared between the two groups using the log-rank test. All statistical analyses were performed using JMP15 (SAS Institute Inc., Cary, NC, USA) and yielded two-sided *P* values. Values of *P* < 0.05 were considered statistically significant.

## Supplementary Information

Supplementary Table

## References

[1] Giulianotti PC (2003). Robotics in general surgery: personal experience in a large community hospital. Arch. Surg..

[2] Kernstine KH, DeArmond DT, Karimi M, Van Natta TL, Campos JC (2004). The robotic, 2-stage, 3-field esophagolymphadenectomy. J. Thorac. Cardiovasc. Surg..

[3] van Hillegersberg R, Boone J, Draaisma WA, Broeders IA, Giezeman MJ, Borel Rinkes IH (2006). First experience with robot-assisted thoracoscopic esophagolymphadenectomy for esophageal cancer. Surg. Endosc..

[4] Boone J (2009). Robot-assisted thoracoscopic oesophagectomy for cancer. Br. J. Surg..

[5] Kim DJ (2010). Thoracoscopic 20 esophagectomy for esophageal cancer: feasibility and safety of robotic assistance in the prone position. J. Thorac. Cardiovasc. Surg..

[6] Puntambekar SP, Rayate N, Joshi S, Agarwal G (2011). Robotic transthoracic esophagectomy in the prone position: experience with 32 patients with esophageal cancer. J. Thorac. Cardiovac. Surg..

[7] Van Der Sluis PC, Ruurda JP, van der Horst S, Goense L, van Hillegersberg R (2018). Learning curve for robot-assisted minimally invasive thoracoscopic esophagectomy: results from 312 cases. Ann. Thorac. Surg..

[8] Suda K (2012). Robot-assisted thoracoscopic lymphadenectomy along the left recurrent laryngeal nerve for esophageal squamous cell carcinoma in the prone position: technical report and short-term outcomes. World J. Surg..

[9] Deng HY (2018). Comparison of short-term outcomes between robot-assisted minimally invasive esophagectomy and video-assisted minimally invasive esophagectomy in treating middle thoracic esophageal cancer. Dis. Esophagus.

[10] Park S (2016). Comparison of robot-assisted esophagectomy and thoracoscopic esophagectomy in esophageal squamous cell carcinoma. J. Thorac. Dis..

[11] Chao YK, Hsieh M, Liu YH, Liu HP (2018). Lymph node evaluation in robot-assisted versus video-assisted thoracoscopic esophagectomy for esophageal squamous cell carcinoma: a propensity-matched analysis. World J. Surg..

[12] Motoyama S (2019). Extensive lymph node dissection around the left laryngeal nerve achieved with robot-assisted rhoracoscopic esophagectomy. Anticancer Res..

[13] Seesing MFJ (2018). Defining pneumonia after esophagectomy for cancer: validation of the Uniform Pneumonia Score in a high volume center in North America. Dis. Esophagus.

[14] Low DE (2015). International consensus on standardization of data collection for complications associated with esophagectomy: Esophagectomy Complications Consensus Group (ECCG). Ann. Surg..

[15] Japan Esophageal Society (2017). Japanese classification of esophageal cancer, 11th edition: part I. Esophagus.

[16] Japan Esophageal Society (2017). Japanese classification of esophageal cancer, 11th edition: part II and III. Esophagus.

[17] Wong IYH, Law S (2016). Surgery in the era of neoadjuvant therapy for cancer of the esophagus. Esophagus.

[18] Ajani JA (2019). Esophageal and esophagogastric junction cancers, version 2.2019, NCCN clinical practice guidelines in oncology. J Natl Compr Cancer Netw.

[19] Lordick F (2016). Oesophageal cancer: ESMO clinical practice guidelines for diagnosis, treatment and follow-up. Ann Oncol.

[20] Kitagawa Y (2019). Esophageal cancer practice guidelines 2017 edited by the Japan Esophageal Society: part 1. Esophagus.

[21] Kitagawa Y (2019). Esophageal cancer practice guidelines 2017 edited by the Japan esophageal society: part 2. Esophagus.

[22] Straatman J (2017). Minimally invasive versus open esophageal resection*: *three-year follow-up of the previously reported randomized controlled trial*:* the TIME. Trial.

[23] Yang Y (2019). Robot-assisted esophagectomy (RAE) versus conventional minimally invasive esophagectomy(MIE) for resectable esophageal squamous cell carcinoma: protocol for a multicenter prospective randomized controlled trial (RAMIE trial, robot-assisted minimally invasive esophagectomy). BMC Cancer.

[24] Yang Y (2020). Short-and mid-term outcomes of robotic versus thoraco-laparoscopic McKeown esophagectomy for squamous cell esophageal cancer: a propensity score-matched study. Dis. Esophagus.

[25] Tagkalos E (2020). Robot-assisted minimally invasive esophagectomy (RAMIE) compared to conventional minimally invasive esophagectomy (MIE) for esophageal cancer: a propensity-matched analysis. Dis. Esophagus.

[26] Harbison GJ, Vossler JD, Yim NH, Murayama KM (2019). Outcomes of robotic versus non-robotic minimally-invasive esophagectomy for esophageal cancer: an American College of Surgeons NSQIP database analysis. Am. J. Surg..

[27] van der Horst S, de Maat MFG, van der Sluis PC, Ruurda JP, van Hillegersberg R (2019). Extended thoracic lymph node dissection in robotic-assisted minimal invasive esophagectomy (RAMIE) for patients with superior mediastinal lymph node metastasis. Ann. Cardiothorac. Surg..

[28] Dindo D, Demartines N, Clavien PA (2004). Classification of surgical complications: a new proposal with evaluation in a cohort of 6336 patients and results of a survey. Ann. Surg..

